# Articulating the “stem cell niche” paradigm through the lens of non-model aquatic invertebrates

**DOI:** 10.1186/s12915-022-01230-5

**Published:** 2022-01-20

**Authors:** P. Martinez, L. Ballarin, A. V. Ereskovsky, E. Gazave, B. Hobmayer, L. Manni, E. Rottinger, S. G. Sprecher, S. Tiozzo, A. Varela-Coelho, B. Rinkevich

**Affiliations:** 1 Departament de Genètica, Microbiologia i Estadística, Universitat de Barcelona, Av. Diagonal 643, 08028 Barcelona, Spain; 2 Institut Català de Recerca i Estudis Avançats (ICREA), Barcelona, Spain; 3 Department of Biology, University of Padova, Via U. Bassi 58/B, 35100 Padova, Italy; 4 Aix Marseille University, Avignon Université, CNRS, IRD, IMBE, Marseille, France; 5 St. Petersburg State University, Biological Faculty, Universitetskaya emb. 7/9, St. Petersburg, 199034 Russia; 6 N. K. Koltzov Institute of Developmental Biology, Russian Academy of Sciences, Vavilova Street 26, Moscow, 119334 Russia; 7 Université de Paris, CNRS, Institut Jacques Monod, F-75006 Paris, France; 8 Department of Zoology and Center of Molecular Biosciences, University of Innsbruck, Technikerstr. 25, 6020 Innsbruck, Austria; 9 Université Côte d’Azur, CNRS, INSERM, Institute for Research on Cancer and Aging, Nice (IRCAN), Nice, France; 10 Université Côte d’Azur, Federative Research Institute – Marine Resources (IFR MARRES), Nice, France; 11 Department of Biology, University of Fribourg, Chemin du Musee 10, 1700 Fribourg, Switzerland; 12 Sorbonne Université, CNRS, Laboratoire de Biologie du Développement de Villefranche-sur-mer (LBDV), Paris, France; 13 ITQB NOVA, Instituto de Tecnologia Química e Biológica António Xavier, Av. da República, 2780-157 Oeiras, Portugal; 14 Israel Oceanography and Limnological Research, National Institute of Oceanography, Tel Shikmona, P.O. Box 8030, 31080 Haifa, Israel

**Keywords:** Adult stem cell (ASCs), Germline stem cells (GSCs), Stem cell niche (SCN), Marine/aquatic organisms, Self-renewal, Phyletic diversity

## Abstract

Stem cells (SCs) in vertebrates typically reside in “stem cell niches” (SCNs), morphologically restricted tissue microenvironments that are important for SC survival and proliferation. SCNs are broadly defined by properties including physical location, but in contrast to vertebrates and other “model” organisms, aquatic invertebrate SCs do not have clearly documented niche outlines or properties. Life strategies such as regeneration or asexual reproduction may have conditioned the niche architectural variability in aquatic or marine animal groups. By both establishing the invertebrates SCNs as independent types, yet allowing inclusiveness among them, the comparative analysis will allow the future functional characterization of SCNs.

## The stem cell niche

Stem cells (SCs) are cells that are able to differentiate into various cell types and are essential for development and homeostasis of multicellular organisms [1–6]. SCs are commonly classified into embryonic and adult SCs (ASCs, also called somatic SCs): both types have the capacity for self-renewal and the ability to differentiate into a series of progenitor cells, though differ in other attributes [7]. In short, embryonic stem cells can be readily grown in culture and exhibit unique properties, including spontaneous differentiation into three germ layers in vitro or teratoma formation in vivo. In contrast, adult stem cells are rare, undifferentiated cells present in many adult tissues. Their primary role is to maintain and repair the tissue in which they reside. The ability of adult stem cells to differentiate is limited.

Tissue-specific SCs in adults reside within compartments called SC niches (SCNs), specific microenvironments that surround SCs and have important regulatory functions in SC survival and proliferation [8]. The concept of the SCN has progressively evolved from its inception [9] and is now broadly characterized by specific morphological properties.

Schofield in 1978 [9] first introduced the idea of the SCN in the context of mammalian hematopoietic lineages as a physical location (a microenvironment) where ASCs reside, receive stimuli, and have their specific fates determined. The concept was soon extended to other tissues and SC types, and eventually, this understanding evolved to a consensus that considers niches as “agents of feedback control” [10]. Other views describe niches as providing “nutritive” (viability-sustaining) functions to tissues [11, 12] or as being involved in the “coordination among tissue compartments” [13, 14]. While multiple subtypes of SCNs may exist (e.g., simple niches, complex niches, storage niches), SCNs are commonly typified by adhesive interactions, cell cycle modifications, and intercellular signals that collectively control ASC statuses [15]. Our current knowledge gap on the cellular, molecular, and system levels in most animal groups has prompted researchers to assume niche-associated properties based solely on circumstantial evidence (i.e., physical location: [16–18]).

The molecular nature of crosstalk within niches (the complex interactions controlled by extracellular cues from the physical niche and by the intrinsic genetic landscapes of SCs [19]) has been characterized in very few contexts, which will be described below.

Marine and aquatic invertebrates offer new opportunities to analyze the structure and function of stem cells, this is due to their specific life strategies, that in many of them include extensive regeneration of body parts or asexual reproduction. Dealing with those processes rely on the fast and widespread mobilization of stem cells. While some of these stem cells have been characterized, a great degree of ambiguity exists in the knowledge of most aquatic invertebrate ASC systems studied to date (Porifera, Annelida, Tunicata, Echinodermata, Cnidaria, etc.), and in many extant research models, the nature and potential of ASCs remain putative and poorly understood (e.g., [5, 20–25]). These putative ASCs are generally characterized by the property of proliferation and/or by the expression of one or more alleged “stemness” markers—for example, PIWI [26–28] (for a critical assessment, see also [16, 29]), PL-10 [30], Vasa [31, 32], and Nanos [33]. While these markers are believed to be associated with germ line development, it has been shown that they are also used in somatic stem cell maintenance [30, 32, 34, 35]. In fact, Alié and collaborators [16] have discussed the role of the “germ line”-specific genes in the more general control of stemness states.

Our main aim in the following sections is to revisit the many definitions that the term SCN has assumed in studies of aquatic invertebrate animals (e.g., sponges, the cnidarian *Hydra*, urochordates, and flatworms (Platyhelminthes) or acoelomorphs). We further focus on the relationships between these definitions and the more rigorously established definition in some of the so-called classical “model” organisms (vertebrates and the ecdysozoans *D. melanogaster* and *C. elegans*). Ultimately, the results of this review enable us to articulate an elaborated “adult stem cell niche” paradigm through the lens of non-model aquatic invertebrates.

## Stem cell niches in vertebrates and ecdysozoan invertebrates

Most of our current knowledge on stem cell systems and their regulatory microenvironments is derived from a few well-studied animal systems (vertebrates and the ecdysozoans *D. melanogaster* and *C. elegans*). These are considered “classical” model systems, with well-developed technologies for gene activity manipulation (briefly described below). For practical and historical reasons, the model systems have been traditionally terrestrial animals [36], and it is for this particular (biased) reason that we start our description of what is known on niches by analyzing these terrestrial models. They should serve us as guides to understand the niches as are being described in aquatic or marine animal systems. For better understanding of the variability of stem cells that are regulated by niches across different metazoan taxa, we refer to Rinkevich and collaborators [5].

### The bone marrow hematopoietic stem cell niche

**Fig. 1:**
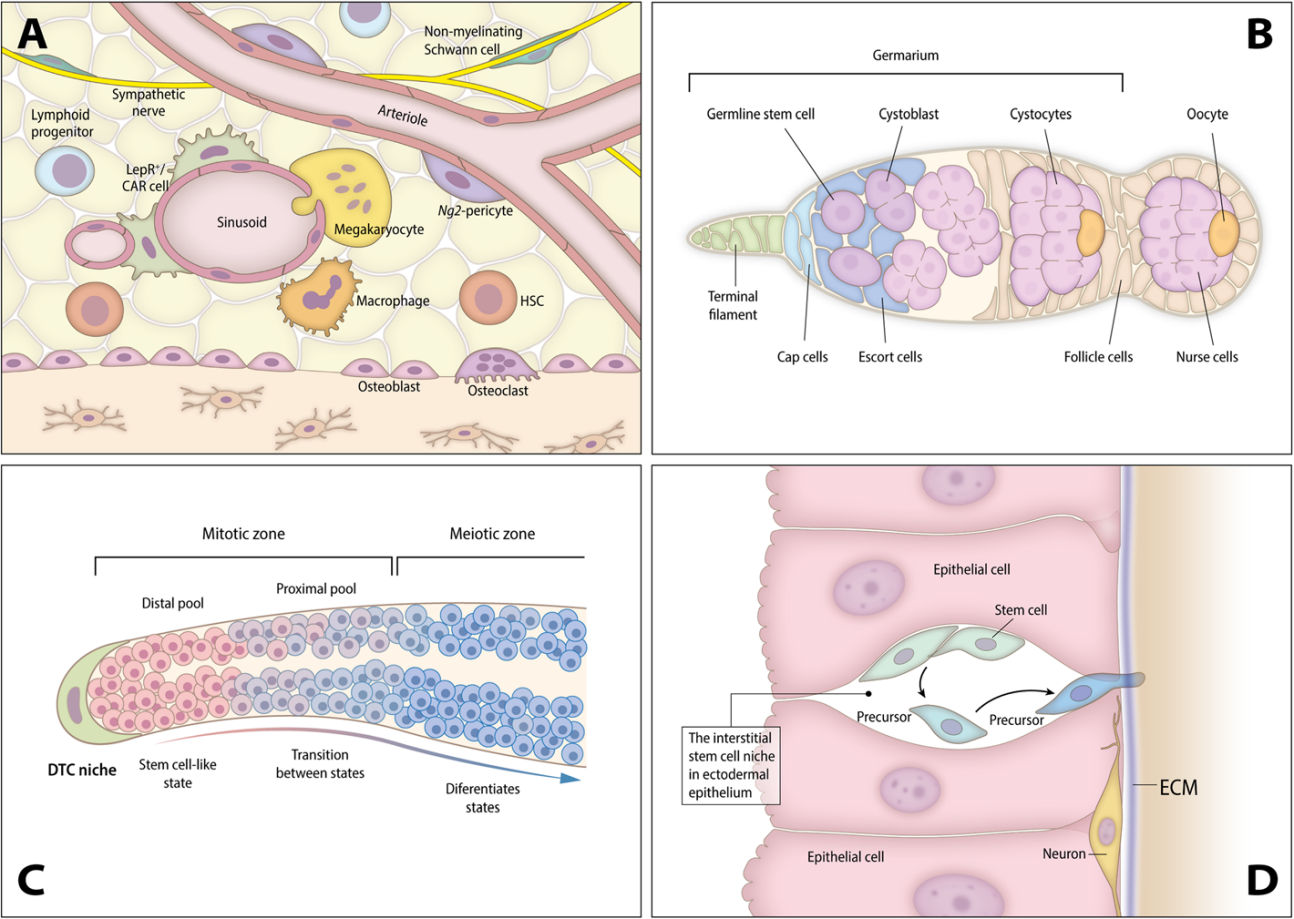
Schematic illustrations for some of the best-characterized stem cell niches. **A** Human hematopoietic SCN: This is one of the best-characterized niches, with the hematopoietic stem cells (HSCs) receiving systemic and local signals. The niche is perivascular, created partly by mesenchymal stromal cells and endothelial cells and often but not always located near trabecular bones (diagram based on [39]). **B** *Drosophila melanogaster* gonadal niche (based on [40]): A germarium with mature oocytes in the proximal region and cap cells in the distant region. The latter cells comprise the major component of the niche and maintain permanent contact with developing germinal cells (here, oocytes, though the sperm cells reside in a similarly constructed niche). **C** *Caenorhabditis elegans* gonadal niche: The germinal line differentiates in a distal-to-proximal direction, with the DTCs (distal tip cells) as key components of the niche (based on [41]). **D** *Hydra* I-cell site location (diagram based on [20]): here a stem cell (I-cell) resides within the epidermal epithelium. Both stem cells and their precursors are maintained via a collection of signals and interactions with the ECM. Precursors not only attach to the ECM but exhibit the capacity to penetrate it and thereby move into the endodermal epithelium. More details on each niche type are provided in the text

Human hematopoiesis is a biological process that produces ca. 200 billion red cells and 10 billion white cells per day per human body. In all mammals, the hematopoiesis of all blood cell lineages is sustained by a rare population of self-renewing hematopoietic stem cells (HSCs). To generate the broad repertoire of white-cell lineages, these HSCs produce an array of oligopotent progenitors, which proliferate and differentiate during static hematopoiesis into mature cells, in a relatively constant manner. Following stress events, such as tissue damage, HSCs commence extensive proliferation and differentiation processes to generate the cells necessary for repair, compensate for blood cell loss, and contain any threat posed by pathogens. These response programs are customarily transient, and within a short time, HSCs return to a quiescent state [37]. HSCs arise during embryonic development and initiate hematopoiesis in specific fetal niches before relocating postnatally to the bone marrow (BM [38];) (Fig. 1A). Several approaches have been used to further characterize the molecular regulators of HSCs (e.g., ex vivo screening for factors supporting HSC maintenance), and the identification of these factors has made it feasible to classify specific cell types involved in the various activities carried out within the niches. In addition to local involvement of different cell types and their mutual interactions, chemical factors that originate at sites distant from HSC niches and the extracellular matrix may participate in modulating HSC and/or niche functions (i.e., [42–44]).

The HSC niche has emerged as one of the best-characterized mammalian SC niches but it may present just a single niche epitome, out of possible three major prototypes (see section “ASCs and their niche: modes of organization” for an in-depth discussion).

### Germ stem cell niches

A notable type of SCN supports germ cells during the lifespan of multicellular animals. Germline SCNs are indispensable for the generation of mature gametes in most sexually reproducing organisms. These germ stem cells and the environments in which they reside have been extensively studied in two model ecdysozoans: the insect *Drosophila melanogaster* and the nematode *Caenorhabditis. elegans*. While the focus of this Review is on adult SCNs, results of studies in these ecdysozoans have provided the basic tenets for the molecular and cellular components of SCNs, some of which are presented below. Other well-described germ cell niches (e.g., the mammalians: [45, 46]) are not discussed here.

#### *Drosophila melanogaster* (Fig. 1B)

Prime examples of well-described SCNs outside vertebrates are the *Drosophila* germline SC (GSC) niches. The first germ SCNs identified in *Drosophila*, anatomically and functionally, were the ovarian niches [47, 48], located throughout pupal and adult life at the tip of each *Drosophila* ovariole (the ovary consists of approximately 16 ovarioles; each with its chain of developing eggs). The *Drosophila* ovary contains two stem cell types: germinal stem cells (GSCs) and follicular stem cells (FSCs). GSCs are located in the germarium, a structure present at the anterior tip of the ovarioles, and are embedded in groups of somatic cells that function as niche cells for the GSCs and FSCs. This morphologically simple ovarian niche consists of three cooperating somatic cell types: terminal filament cells (TFs), cap cells (CpCs), and escort cells (ECs, or inner germarium sheath cells; Fig. 1B). Two to three GSCs are directly associated with CpCs. GSC divisions within the niche are usually asymmetric as only one of the daughter cells remains in the niche while the other differentiates into a cytoblast. FSCs are located more posteriorly in the germarium. They are closely associated with a type of EC (posterior ECs, or PECs) and are the source of both ECs and all somatic cells located posteriorly to the FSCs, including the follicle cells surrounding germline cysts [49]. In the GSC niche, key regulators are the molecules Dpp, abd, Hedgehog, and components of the extracellular matrix [49–51].

The male SCN morphologically resembles the architecture of the ovary, as stromal cells at the distal tip of the testes maintain intimate contact with the GSCs. The advent of single-cell sequencing technologies has recently allowed the generation of comprehensive cell atlases of both *Drosophila* ovaries [52–54] and testes [55]. These analyses include extensive transcriptional profiles of all major cell types present in the gonads, including the germline stem cells and their surrounding niche cells. The contributions of these studies are beyond the scope of this review.

#### *Caenorhabditis elegans* (Fig. 1C)

GSCs are the only bona fide SCs in the nematode [56, 57]. Laser ablation experiments on cells in the *C. elegans* gonads [58] were the first to suggest that extrinsic cues may play a role in controlling different lineage decisions. These experiments were also the first to determine interactions between neighboring cells (the “niche”), establishing germline specification. In this hermaphrodite worm, the SCs reside in the blind-ended tubes of the gonads, and in GSC niches, each tube sustains a single mesenchymal niche cell called the distal tip cell (DTC [58]; Fig. 1C). The DTC (Fig. 1C) provides an elaborate “plexus” of cellular processes that enwraps approximately 10 GSCs, increasing intimate contact between the SCs and the niche cells. The DTCs are essential for the maintenance of GSC stemness and “allow” the maintenance of hundreds of dividing cells within a so-called “mitotic and proliferation regions.” DTC regulates the balance of self-renewal and early differentiation within the “mitotic region” [59]. The major component of this molecular circuitry is the GLP-1/Notch signaling pathway, a broadly conserved pathway within the Metazoa [60, 61] that also regulates niche–SC interactions in mammals (e.g., in the nervous system, muscle, intestine, skin, and hematopoietic system) (e.g., [62, 63]). This architecture of the *C. elegans* GSC niche, with GSCs at one end and progressively differentiating cells nearer the open end, appears similar to the GSC niches in the *Drosophila* male and female GSC niches. The uncovering of cell protrusions mediating the communication within the stem cell niche adds to the structural similarities between *C. elegans* and *Drosophila* gonadal niches [64, 65]; similar structures were recorded in the gonads of a milkweed bug [66]. As in *Drosophila*, new technologies that allow the construction of expression maps with single-cell resolution (e.g., transcriptome profiling [67] or RNA tomography [68]) have been extremely useful in dissecting the multiple components that regulate the cellular interactions within the GSC niche.

### A unified view for SCN (Fig. 2)

**Fig. 2:**
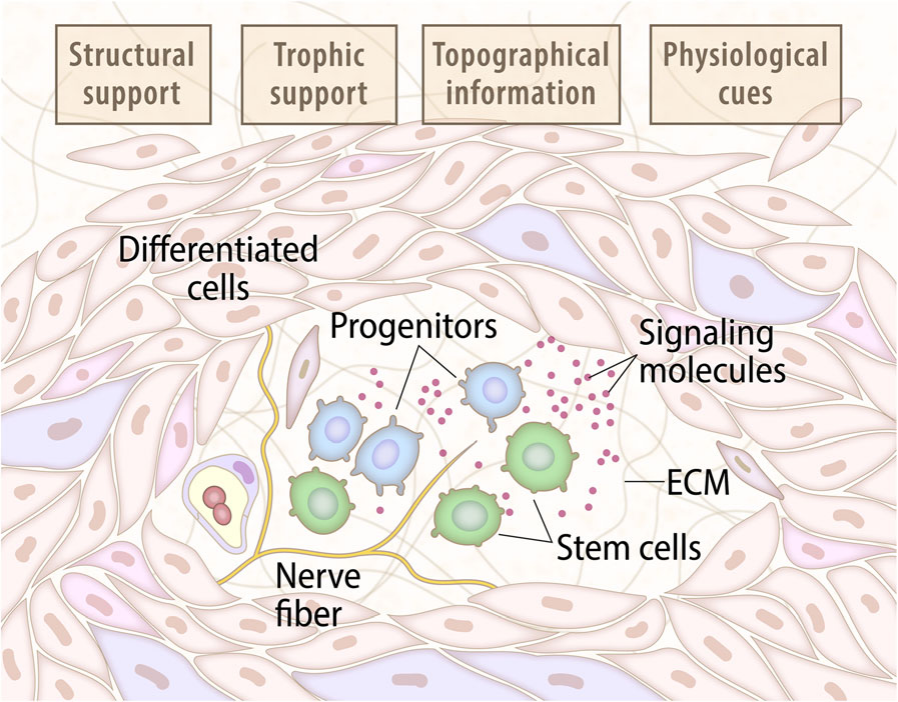
Unified current view for the structure and properties of the niche in model organisms. This illustration presents the most significant basic structures assumed to define a stem cell niche, as derived from studies of vertebrates’ hematopoietic systems (and of other SCNs in various organs) and the germ cell niches of the non-ecdysozoan model invertebrates *Drosophila melanogaster* and *Caenorhabditis elegans*. At the top, we list the four physiological properties associated with niche functionality (derived from [69]). The different cellular, signaling, and matrix components affiliated with SCN activities are further depicted (based on [70]). Variations in the presence of the different components occur in different animal systems (and may also be explained by a current lack of knowledge)

The research conducted on the “model” organisms above offers a general conceptual view of the unified structure and properties associated with SCNs (Fig. 2). Four classes of physiological properties are associated with all niche functionalities: structural support, trophic support, topographical information, and physiological cues [19, 69, 71]. In brief, we have learned from the so-called “model systems” that the stem cell niche refers to a group of cells in a special tissue location devoted to the maintenance of stem cells. The niche’s overall structure is variable, and different cell types plus molecular regulators can provide the niche environment [19]. The formation and activity of niches (at discrete developmental times) are carefully regulated to ensure appropriate stem cell function. Because stem cells can function either homeostatically (continuously replacing short-lived mature cells that are lost because of normal cell turnover) or facultatively (replacing differentiated cells only in response to injury or disease), stem cell niches must be dynamic enough to provide proper developmental and physiological cues to regulate stem cell behavior. This role implies the regular mobilization of stem cell activity in response to environmental (physiological) conditions [69]. The niches provide structural and functional cues that are both biochemical and biophysical, and the SCs integrate this complex array of signals with intrinsic regulatory networks to meet physiological demands. These SCs are supported by, or incorporated into, the niche walls formed by the neighboring cells; hence, SC functions rely on geometric cues that orchestrate several niche-associated mechanochemical and paracrine-autocrine signals, which they can direct to acquire appropriate fates. Subtle shape cues can also play a significant role in promoting differentiation [71]. It becomes clear then that, in this context, the niche itself, situated in specifically designated sites [19, 69], assumes a distinct and well-defined morphological structure that is composed of four cellular components (SCs, progenitors, differentiated cells, and nerve fibers) as well as the ECM and the signaling molecules that are governed by the four classes of physiological properties. The coherent integration of all components (Fig. 2) is critical for the proper functionality of the SCN. The above considerations guide us in our exploration of SCNs, which, while focusing on marine/aquatic animal systems, considers a wider taxonomic range.

## Stem cell niches in aquatic and marine invertebrate systems

Although the majority of known species are terrestrial, all higher animal taxa originated in aquatic (marine and freshwater) environments, and most of the extant animal phyletic diversity is still found there [72–74]. In recent decades, several studies have suggested the existence of SCNs in a handful of aquatic invertebrate systems [18, 24, 75–79]. In contrast to the vertebrate and mostly ecdysozoan invertebrate models, however, data supporting these proposed SCNs are fragmented and sometimes elusive. While the following examples of ASC niches in marine invertebrates underscore the paucity of these data, they also uncover some novel evidence supporting the role and possibly the overall conservation of general architectures within different clades. Needless to say, overall conservation does not prevent niches from showing variability.

### Cnidarians: *Hydra* and *Hydractinia*

Among cnidarians (sea anemones, corals, and jellyfish), the hydrozoans *Hydra* [80] and *Hydractinia* (a colonial hydrozoan [81]) are the best-studied research models for ASC biology (Fig. 1D), and a *Hydra* SCN has previously been proposed [20, 77]. Investigation of ASCs and SCNs in anthozoan cnidarians (sea anemones, corals) are only in their infancy [82, 83]. *Hydra* and *Hydractinia* ASCs consist of three distinct ASC lineages. Two ASC lineages, epidermal and gastrodermal epithelial stem cells, are epitheliomuscular cells that construct and maintain the simple two-layered (epidermal and gastrodermal) body of the polyps. The third multipotent interstitial ASC lineage (the interstitial cells, or I-cells) produces a variety of differentiated cell types, including somatic neurons, nematocytes (stinging cells), and gland cells as well as the gametes [84, 85]. In *Hydra,* these three SC lineages contribute to a highly dynamic state of permanent tissue replacement via self-renewal and differentiation, which is the basis for the polyps’ extreme longevity and capacity for continuous budding and regeneration. Transdifferentiation of a cell from one lineage to another (commonly recorded in jellyfish [86]) does not occur in *Hydra*. However, interstitial SCs can generate new epithelial cells following allogeneic colony fusions in *Hydractinia*, suggesting that they harbor an even larger degree of potency compared to those in *Hydra* [87, 88].

Hydrozoan I-cells reside in compartmental caverns between epithelial cells, located in *Hydra* within the epidermal layer and throughout the entire gastric region of the polyp [89, 90] (Fig. 1D). By directing the maintenance of the multipotent I-cell state and the commitment to become a precursor cell, these compartments were presumed to carry the functions of a complex SCN entity [77]. However, the mechanisms (molecular players), including short- and long-range signaling factors acting in the SCN have not been elucidated. Light and electron microscopy studies have revealed that I-cells are in intimate contact with the surrounding ectodermal epithelial cells over almost their entire membrane surface, while no unambiguous proof has been offered for direct contact with the mesoglea, the polyp’s ECM (Fig. 1D [20, 91];). The proteins involved in direct epithelial–interstitial cell membrane interactions are unknown, but *Hydra* classic cadherin is a strong candidate due to its expression in the epidermal epithelium and in the I-cells (B. Hobmayer, unpublished). In addition, the transcription factors *Myc, FoxO*, and PIWI proteins are engaged in I-cell maintenance, and Notch signaling seems to promote differentiation of nematocyte precursor cells [27, 92–94]. A search for specific multipotency and maintenance factors in the putative I-cells pool using in-depth single-cell transcriptome sequencing has identified only a single gene, *hy-icell 1*, whose corresponding protein sequence does not show similarity to any known protein family, and yet its precise function is unclear [95]. However, a core set of I-cells marker genes could not be identified, and the study concluded that I-cells may primarily be defined by the absence of differentiation gene modules [95]. The commitment of I-cells toward differentiation into nerve or gland cells of the endodermal layer prompts the resulting precursors to establish contact with the mesoglea (Fig. 1D), to migrate through this milieu by as yet unknown mechanisms, and to enter caverns in the inner epithelium where they finally undergo terminal differentiation. *Hydra* I-cells are more stationary, but they nevertheless possess the capacity to migrate to “empty” caverns when confronted with an ectoderm devoid of I-cells [92], and they adapt their ability for self-renewal in response to changes in I-cell and nerve cell densities. These results imply a significant role for long-range signaling input in the activities of the niche [96]. As in many bilaterians, Wnt/β-Catenin signaling likely serves as an important cue in *Hydra* and *Hydractinia* [97, 98]. Originating from the polyp’s major signaling center in the head, this pathway functions as an upstream regulator of Myc activity enhances I-cell maintenance and affects the probabilities of nematocyte and nerve cell differentiation [99–101]. Holstein and David in 1990 [102] also showed that precursor cells from the foot-peduncle region of a *Hydra* that are committed for neuronal differentiation, can be forced to revert and become interstitial stem cells again, suggesting a rather direct impact of global patterning signals on interstitial stem cell potency. Further, interstitial stem cell localization (central body column), nerve cell differentiation (head and foot areas), and nematocyte differentiation (central body column), all follow axial positional values [100] rather than SCN properties. Taken together, the above definition of the niche—with distinct physical components and chemical interactions—implies its involvement in the decision-making process of interstitial SCs.

The epidermal and gastrodermal epithelial ASCs in *Hydra* pose a further challenge to SCN forbearance in cnidarians as no experimental evidence depicts the existence of distinct subpopulations with different levels of stemness [77, 91, 103]. These ASCs concurrently satisfy the criteria for an SC and act as differentiated cells; thus, stem cells, differentiated cells, and putative niche cells are no longer distinct from one another. Under these conditions, it is difficult to imagine a physically and chemically specific niche at the level of a single epithelial cell. Attempting to address this dilemma, Bosch and colleagues [77] speculated that the entire epithelium behaves as a single global epithelial SCN due to the ability of all cells to maintain proliferation and differentiation states.

While cnidarian model polyps such as *Hydra* and *Hydractinia* are powerful models for understanding animal regeneration, some scyphozoan (jellyfish) species (which lack I-cells) are interesting reference cases for the study of adult body’s regeneration [104]. Similarly to the Scyphozoa, anthozoans (e.g., sea anemones, corals) also lack hold I-cells, and while some studies (i.e., [105]) have recognized aggregates of stem cell-like structures in fast growing tissue layers, neither a putative stem cell type has been described morphologically nor an SCN like structure was documented [106].

The recent development of techniques to manipulate gene expression in hydrozoans [107–109] suggest the possibility of analyzing the molecular regulators of many processes in which stem cells and their niches are involved. By dissecting the different molecular players and their physical locations, we should gain insights on how the SCN functions, enriching our understanding of the configuration and variability of niches across the Cnidaria.

### Platyhelminthes and Acoelomorpha

Several species of Platyhelminthes and Acoelomorpha (acoel flatworms) exhibit extensive regenerative capacities, up to whole-body regeneration [110–114]. This capacity requires a group of ASCs called neoblasts [115–117] that are located within the parenchymal tissue of many free-living (e.g., [111, 118]) and parasitic flatworms [119]. In well-studied species, such as *Schmidtea mediterranea*, the neoblasts constitute up to one-third of the animal’s cells [120] and are known to be the only proliferating cells in the adult body. Regeneration properties have been thoroughly examined in a few species: the Platyhelminthes *S. mediterranea* [111, 121], *Dugesia japonica* [122], the tapeworm *Hymenolepis diminuta* [114], and the acoels *Isodiametra pulchra* [26] and *Hofstenia miamia* [123, 124]. Early experiments demonstrated that all new somatic cells emerging during tissue maintenance, as well as during regeneration, originated from neoblasts [111, 125]. Upon transplantation into irradiated animals, neoblasts (even a single neoblast in Platyhelminthes) were shown to be able to replenish all tissues in *S. mediterranea* [111], an experiment not done yet in acoels. In both clades, neoblasts are characterized by their low cytoplasm-to-nucleus ratio; furthermore, their body location and gene expression patterns are remarkably similar in these two phylogenetically distant clades [124]. The observation that even a single cNeoblast (clonogenic neoblast) in lethally irradiated planarians is enough for complete body regeneration, suggests that the environment that supports neoblast proliferation and specification (niche?) is spread over large areas of the animal’s body [126]. In this study of Wagner and collaborators injections of single cNeoblasts were done into the post pharyngeal parenchyma, a relatively broad area, in conjunction with other gene knockdown experiments (see below), all suggesting that the “niche” occupies most of the animal body.

For decades, scholars have recognized neoblasts as a mixed population of cells with different potencies, and in 2011, Wagner and collaborators [126] first demonstrated the existence of a subpopulation of neoblasts in *S. mediterranea*, called clonogenic neoblasts (cNeoblasts), that are genuinely pluripotent. Yet, the distribution of the cNeoblasts within the animal’s body remains to be elucidated, as well as the mechanisms regulating cNeoblasts pluripotency, even though the expression of conserved genes regulating pluripotency have been identified [127–129]. Initially identified as *smedwi-1*-positive cells, the cNeoblast were detected on a body-wide distribution [126]. Studies further revealed that cNeoblasts can be molecularly characterized as expressing the cell surface protein tetraspanin-1^+^ (TSPAN-1^+^). Singly transplanted TSPAN-1^+^ cells rescued lethally irradiated planarians at a much higher frequency than was previously reported [130]. Neoblasts may also switch their specification state and generate a cohort of daughter cells with different fates [131], explaining the rapid regeneration of planarians (however, these authors show here that tetraspanin-1 is not exclusive to the putative planarian cNeoblasts). Attempts at culturing neoblasts in vitro have been developed over the years culminating in some recent promising methodologies (Lei et al., 2019; Biorxiv, preprint). A series of papers have suggested the role of extrinsic factors in the control of neoblast specification, such as the roles of intestinal cells and the homeobox-containing gene nkx-2.2 in regulating neoblasts’ proliferation [132], the roles of ECM surrounding the gut [133], regulation of gap junctions [134], and the roles of some neural neuropeptides [135]. The proximity of the neoblast populations to the gut [130] may endorse the opinion for the intestine as the best candidate tissue that provides niche signals in planarians. In fact, the injection of new altered cells (manipulated egfr-1/ngr-1 cells) to the gut branches leads to a decrease in the proliferation of neoblasts; irrespective of the location of the gut branches [136]. Further, the presence of progenitors of some cell types (such as eyes) in large (yet not all) body areas [137] suggests inherent variability in the global cell niche distributions. In acoels, the neoblast system has been less explored. While in these organisms, neoblasts likewise replenish tissues after irradiation, studies have yet to prove the existence of cNeoblasts [26, 138]. Acoel neoblasts can generate a mixture of mature cell types [138], but whether these lineages are derived from lineage-restricted SCs and their progenitors or a pool of pluripotent SCs remains to be determined. Single-cell transcriptomics data have led to the recognition of specific signatures for neoblast and neoblast-like cells [139], pointing to the possibility that neoblast differentiation trajectories could be deciphered.

Despite advances in our understanding of Platyhelminthes and acoelomorph SCs and regeneration, very little is understood about specific SCNs or signals from differentiated tissues that might impact neoblast stemness and fate [112, 140]. Neoblasts are embedded in the central parenchyma of the animal with no known structural boundary (like a niche) to secure them from other cells [141]. This has led, on the one hand, to the suggestion that the entire animal can be regarded not as an individual but as an assemblage of globally regulated ASCs, in what can be equated to a single whole-body SCN [142, 143] and, on the other hand, to proposals to designate compartments within the parenchyma as SCNs [78]. The fact that planarians are able to regulate homeostasis at the organism level [144] seems to favor the presence of widely distributed control mechanisms. The different lines of evidence mentioned in this section suggest the involvement of specific cell types, signaling factors, and the ECM in configurating the planarian niche. However, as these are still indirect pieces of information, a comprehensive and detailed description of niches in the Platyhelminthes—and certainly in the Acoelomorpha—remains to be elucidated. Specific data on the regulatory mechanisms that control the proliferation/differentiation of single cNeoblasts would be particularly enlightening [75]. Flatworms also possess niches around the germline—this is out of scope for the current review but see [145, 146] for more information. A final point here is that planarians have no sequestered germ line so the niche components might be shared with the rest of the body.

### Tunicates

The potential existence of SCNs in invertebrate deuterostomes has been investigated in only a few tunicate species, primarily in the colonial ascidian *Botryllus schlosseri.* In addition to sexual reproduction, *B. schlosseri* propagates asexually, forming colonies of genetically identical modules, or zooids, that are connected via a ramified blood system [147, 148]. While the cellular origin of budding processes remains to be elucidated, the presence of putative ASCs in this process has been proposed [22]. In vivo cell labeling, cell engraftment, and time-lapse imaging have identified two sites of putative ephemeral SCNs in *B. schlosseri* zooids that are replaced weekly. The first are the anterior ventral regions of the subendostylar sinuses, which have been suggested to harbor and export putative somatic SCs, while the second are the cell islands located in the ventral body wall, along the endostyle, which have been suggested to harbor putative GSCs [24, 25, 32, 149]. The endostyle area (the long glandular groove in the ventral side of the zooid’s branchial sac) in tunicates is considered the invertebrate chordate homolog of the vertebrate thyroid gland [150]. Yet it was found that the zooid and bud endostyles express β-catenin, PIWI, Oct4, among others, suggesting also a role as a SCN for bud development [24, 25, 151], where it was suggested that ASCs compete for locations within developing SCNs [152]. Moreover, the endostyle putative niche is considered comparable to the hematopoietic niche [149], as it shares with the human hematopoietic bone marrow the expression of more than 300 genes and harbors cell populations expressing genes characterizing vertebrate hematopoietic stem cells. For the presumed germ line niches, Rosner and collaborators [34] have described a novel network of several transient sites that preserve primordial germ cell (PGC) homeostasis, potentially protecting these cells from the weekly senescence processes occurring in botryllid ascidians and thus enabling the survival of the PGCs throughout the organism’s life.

Colonial ascidians belonging to the genus *Botrylloides* are known for their whole-body regeneration (WBR) phenomenon, in which the entire colony regenerates from only a small portion of the vasculature [153–155]. Recently, Kassmer and collaborators [154] identified a population of bloodborne candidate SCs responsible for WBR in the species *Botryllus schlosseri.* These integrin-alpha-6-positive (Ia6+) cells, which constantly divide in healthy colonies, also express genes associated with pluripotency. During WBR, Ia6+ cells reside in receptacles of the left vasculature, and the beginning of the regenerative process seems to be regulated by Notch and Wnt signals. Additional studies in *Botrylloides* regeneration niches are needed to further understand the relationship between the vasculature pouches and the behavior of Ia6+ candidate SCs.

In *Ciona intestinalis*, a model solitary ascidian, the amputation of the oral siphon triggers the proliferation and migration of cells from nearby regions (short-distance regeneration) and more distant regions of the branchial sac (long-distance regeneration). Short-distance regeneration does not seem to require cell proliferation; rather, it relies on small aggregates of stem/progenitor cells that are already present in the siphonal area [156, 157]. In contrast, long-distance regeneration requires the activity of stem/progenitor cells that originate in regions of the pharynx known as lymph nodes or “ASC niches” [158]. These putative SCNs are located in the enmeshed transverse vessels within the branchial sac. Progenitor migrating cells that express PIWI are believed not only to have roles in homeostasis but also to contribute to wound healing and the regeneration of the oral siphon tissues and the central nervous system [157, 158]. Recently, the presence of proliferating putative ASCs expressing PIWI and high activity of aldehyde dehydrogenase have been reported in the intestinal submucosa of *Styela plicata* (another solitary species), which suggests the existence of a potential niche [159].

While all of the studies detailed in this section have identified specific regions of the body where putative ascidians SCs are maintained and activated during regeneration, budding, and homeostasis, almost nothing is known about the cytoarchitecture of these presumptive SCNs, the nature of the signaling pathways within the putative resident SCs, or even the existence of the regulatory signals.

## Do stem cell niches exist among other marine invertebrates?

In the aquatic invertebrates discussed in the previous sections, the term “stem cell niche” has often been employed loosely without the supporting evidence that should accompany a properly described niche. This shortcoming is further highlighted in cases where much less is known regarding SC biology and SC residence. In the Demospongiae, one main lineage of sponges (Porifera), decision making of the ASCs, such as the amoeboid archaeocytes migrating within the mesohyl, is not associated with any known particular microenvironment [21, 160]. However, in the symplasmic Hexactinellida lineage, archaeocytes are frequently attached to one another and to the trabecular syncytium by plugged junctions, forming clusters or congeries [161]. Because archaeocytes in congeries (clusters, as in Ijima, 1901 [162]) present a high number of mitotic figures, Singla and Mackie (1983) have suggested that each congery may present a mitotic progeny [163]. Nevertheless, any similarity of these structures to the niche concept remains, at present, too preliminary. Another ASC lineage in sponges are the choanocytes, the progenitors of both somatic cells and gametes and the most actively proliferating cell type in these animals [164]. Choanocytes are specialized epithelial cells, responsible for the movement of water inside the sponge’s aquiferous system and for the capture of food particles. Choanocytes are organized into choanocyte chambers, or tubes in asconoid sponges [165]. Individual choanocytes can leave the choanocyte chamber and transdifferentiate into germ or somatic cells. In the case of spermatogenesis, the entire choanocyte chamber is isolated and completely transformed into spermatic cysts [166] but without any support from a SCN.

In corals (Cnidaria), where ASCs are not defined (I-cells do not exist in the Anthozoa), Raz-Bahat and collaborators [106] identified disorganized aggregations of cells with distinct nuclei (putative SCs) in rapidly developing areas, but no morphological distinction for putative SCN has been offered. In the sea anemone *Nematostella vectensis*, two potential ASC populations have been identified; one fast-cycling population enriched in the body-wall epithelia and a slow-cycling population that is enriched within the mesenteries [167]. While additional work is required to confirm the potency of these ASC as well as their molecular signatures, the presence/characteristics of one or several SCNs in anthozoans has to be investigated. Other cases are the ctenophore *Pleurobrachia pileus* [16] and the annelid *Capitella teleta* [18], where the term “niche” has been applied to the anatomical areas where the putative SCs reside (in the tentacle bulbs and coelomic cavity of the 5th segment, respectively). However, no evidence suggests that these are totipotent or multipotent SCs; rather, in some cases, the identified SCs are committed progenitors dedicated to specific lineages (e.g., in *Capitella*, the germline). Moreover, when tentacle bulbs of the ctenophore *Mnemiopsis leidyi* were eliminated, null impacts in regeneration capacities were revealed [168]. In all of the latter cases, the still circumstantial evidence suggest that scientists should be more careful using of the term “niche” to that place where a stem-like cell is located.

At this stage and following the generalized view for the structure and properties of SCN gathered through the studies in model organisms, we propose a comprehensive set of essential parameters associated (or required in) with the SCN: (a) the clear identification of residing stem cells, (b) a detailed map of the cellular components and the ECM, (c) evidence of molecular crosstalk between stem cells and surrounding environmental components, and (d) functional assays (depletion or transplantation). Without a comprehensive assessment of these properties, any proposed SCN should remain a speculative “entity” in whatever biological system it is studied.

## ASCs and their niche: modes of organization

**Fig. 3:**
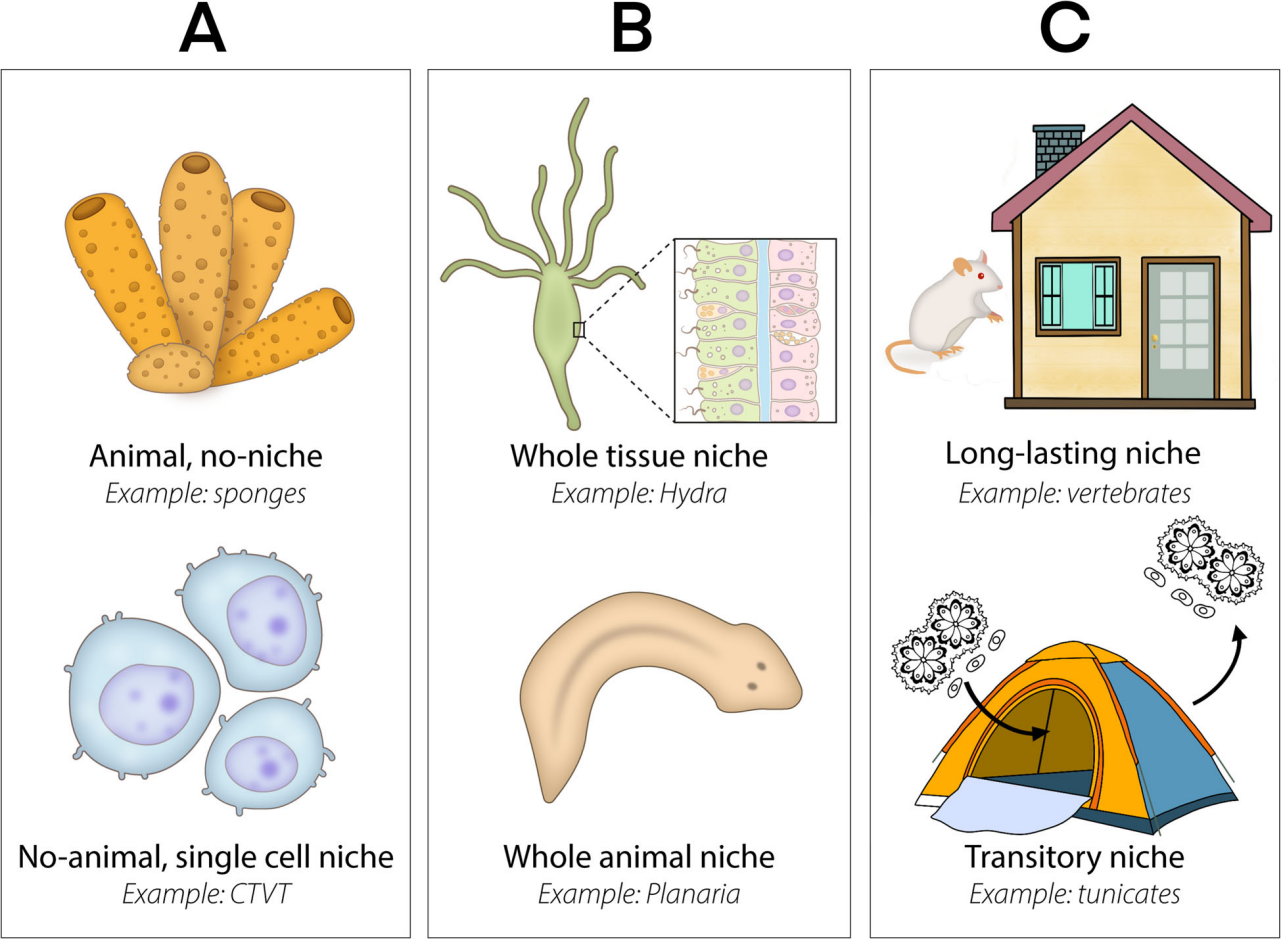
A conceptual ideograph representing the three distinct architectures for the stem cell niche notion in metazoans. **A, B**, and **C** refer to three structurally defined states assigned to describe the progressively complex architecture of niches and their cognate locations in an animal’s body. State **A** represents statuses with no connatural niche, where individual cells bear stem cell properties in their own existence, and cell fates are regulated through interactions with abutting cells. A cellular example that fills this criterion is: CTVT = canine transmissible venereal tumor. State **B** extends conceptual niches to the level of the whole tissue or the whole animal, which, by abductive reasoning, provide the appropriate habitat and foundation for the numerous SCs that reside and proliferate within a permanently existing niche holograph. The State **C** prototype epitomizes the well-structured and enduring model SCNs (typical of vertebrates) along with cases of ephemeral SCNs. Transitory niches = niches that are functional for a short period of time (about 1 or 2 weeks in botryllid ascidians), prior to ASCs departing the SCN—in concert with SCN degradation—and moving through vasculature to newly developed SCNs. Note that although sponges are presented here within the state A category, different clades bear state A and B architectures

The comparative analyses of SCNs summarized above and in the existing literature suggest the presence of three types of SCN states among metazoan animals, categorized as states A, B, and C (see Fig. 3). While we assume that these categories distinctively represent specific properties (Table 1), this does not imply that all niches have to be shoehorned into these three states. In fact, the conundrum of niche architectures is, as we suggest, shaped by natural selection forces that amalgamate niche architectures and constructions into a few general archetypes, all situated on a continuum of structural/functional properties, from loose cells, with each carrying its own niche belongings, to the most complex and structurally defined alcoves (Table 1; Fig. 3). Yet, each one of the suggested SCN states represents the construction of a distinct level of biological organization and all SCN states embrace, under a single conceptual SCN notion, an evolutionary highly evolved assembly.

**Table 1:** Traits and properties assigned to the three distinct niche states

Property/trait	A	B	C
Structural support	No, ASCs are niche-like independent entities	No, whole tissue/organism consideration	Yes, spatially confined
Maintenance and regulation of ASCs	At the level of each specific ASC	Loosely- on the whole organ/tissue/organism level	Tight
Cell-cell interactions	Stochastic, local interactions	Stochastic- on the whole organ/tissue/organism level	ASCs interact with specific niche cells (adhesive interactions)
Cell- environment interactions	Indistinguishable between somatic cells and ASCs	Stochastic- on the whole organ/tissue/organism level	ASCs interact with specific cues from the environment
Physiological cues	Indistinguishable between somatic cells and ASCs	Stochastic- on the whole organ/tissue/organism level	Yes
Trophic support	No	Unknown	Yes
topographical features	No	No	Yes
Niche functionality	No	No	Yes
ASC fates	Independent to any specific microenvironment	Independent to any specific microenvironment	Linked with their homing niche
ASCs- cell cycle modifications	Stochastic	Stochastic	Specified to niches
Regulation	At the ASC level	At the entire organism/tissue levels	At the niche level
Peak potency of ASCs	Totipotency and pluripotency	Totipotency and pluripotency	Multipotency (few cases of pluripotency)

The A (*no obvious niche*) architecture applies to animals that do not possess structured niches. Such animals carry either highly plastic ASC repertoires, i.e., ASCs appear when needed (e.g., the mesenchymal archaeocytes of some poriferans), or with each SC creating their own intimate unstructured environment, in lieu of a shaped niche. The latter, although not applicable for healthy/homeostatic tissue of vertebrates, is speculated to be the case for various allogeneic cancers—naturally occurring transmissible, super-parasitic cell lines that form in vivo chimeras [169]—such as the canine transmissible venereal tumor, the Tasmanian devil facial tumor disease, the clam leukemia [170], and for the cancer SCs that form specialized permissive microenvironments along the tumorigenic cascade [171–176]. These allogeneic tumor cells and cancer SCs are maintained and perform their biological functions within the context of environmental niches (sensu Di Santo, 2008 [177]), or by colonizing ‘new anatomical niches’ (sensu Ayala-Díaz and collaborators in 2017 [178]), altogether creating another form of A SCN state.

Among aquatic invertebrates, the best-known case of SCs residing within A-type niche states is the multifunctional mesenchymal archaeocytes of the Demospongiae, which are not limited, in terms of functionality or fate, by their microenvironment, though they may be regulated by an autocrine system. Yet, there is no conclusive evidence that archaeocytes associate with any type of niche, morphologically or molecularly, taking also their high mobility and the observation that they do not form cell aggregates. Okamoto and collaborators [173] developed this latter proposition by stating that “archaeocytes might be able to stay in an undifferentiated state by using autocrine signals. Only when the inductive signals over a threshold level are received would archaeocytes become committed archaeocytes”. The fact that some sponges can regenerate their entire body from dissociated and reaggregated cells also suggests the lack of a microenvironment providing signals or structural elements. In fact, the initial stages of regeneration (perimorphs) are characterized by the extensive transdifferentiation and reorganization of cell types [179–181], suggesting that morphogenetic processes are most likely guided via local cell-to-cell interactions without the need for a spatially structured “niche domain.” Also, the migratory nature of archaeocytes seems incompatible with the need for localized signaling areas. The B state architecture applies to animals that exhibit distinctive groups of ASCs distributed across their body, even though they are not distributed evenly everywhere in the animal nor exhibit regionalized gene expression (e.g., Forsthoefel et al. 2020). These statuses echo in a larger scale the statuses within “classical” SCNs (e.g., [182, 183]). Animals harboring B niche states either contain a whole organ-tissue SCN, like suggested in *Hydra* [77], or the whole animal acts as niche, i.e., the case of Platyhelminthes [142, 143]; yet, understanding the nature of the niche in planarians is still a work in progress. B states may also be inferred in the sponge choanocytes, as the choanoderm epithelium in the aquiferous systems of different sponge groups is the microenvironment supporting choanocyte proliferation and differentiation [184, 185], resembling the *Hydra* status. Thus, different classes of a phylum (e.g., Porifera) may present state A, B, or both A and B SCN states within their body. It should also be noted that recent research on cell atlases from various invertebrate taxa, including the demosponge *Spongilla lacustris* [186], *Hydra* [95], the hermatypic coral *Stylophora pistillata* [187], the planarian *Schmidtea mediterranea* [188], and the trematode *Schistosoma mansoni* [189], while revealing broad and detailed lists of cell types within each studied invertebrate species, did not elucidate any cell type that was suggested nor assigned to a stem cell niche, further supporting the notion for the absence of “classical” stem cell niches in states A and B, in organisms that differ in several key characters (Table 2).

**Table 2:** Key characteristics distinguishing A and B SCN states

Character	State A	State B
*Stemness*	Pluripotent at most	Totipotent (developing the soma/germ cell lineages).
*Regulation*	At the SC level	At the entire organism/whole tissue levels
*Niche architecture*	No. niche. Each ASC may sustain its own private self-regulation intimate environment	The entire organism/whole tissue constructions
*Level of molecular pursuit*	Self-regulating (cancer cells, archaeocytes, choanocytes)	Systemic
*Motility of cells*	Highly motile	Restricted motility
*Distribution*	Random (archaeocytes, cancer cells)	More ordered in the animal milieu (sponge choanocytes, pinacocytes, I-cells in Hydra, neoblasts in flatworms)

The third class of a niche state (state C) is present in those animals that possess spatially confined (and interaction-rich) ASC niches, primarily mammals and insects [190–192] and possibly in transient putative SCNs of some tunicate species [24]. In those cases, spatially confined ASCs and transient SCNs niches, their composition, relative distribution of cells, and the extracellular matrix are fundamental to ensure both the maintenance of the stem cell stage and the regulation of their differentiation (in time and response to specific needs). Some clear examples have been described in tissues such as the gut, epithelium, or neural, where specific pools of tissue-restricted stem cells supply cells for renewal or homeostasis (for details, e.g., [4]). Here, the cells in the SCNs interact with the ASCs through local and systemic regulators (e.g., hormones), which introduce an additional level of complexity. Regulation of stem cells (somatic or germinal) by hormones has been reported in few marine/aquatic invertebrates, notably the annelid *Platynereis dumerilii* [193, 194]. These three types of SCN states are most likely linked to the different nature of the stem cells in each animal group. Processes such as regeneration or sexual reproduction have a clear impact on the way that stem cells and their microenvironments are constructed and regulated. The increasing complexity of niches (from states A to C) may reflect different levels of constraints or greater lineage flexibility in the ASCs supported by these microenvironments.

The three different niche states (Fig. 3) have been assigned based on the current literature, further expanding the SCN concept for different contexts and animals, including the model organisms. The C state, presents as a well-structured SCN (e.g., [56, 195, 196]), is the perfect domicile for tightly regulated stemness statuses where stem cells may home and undergo education processes, such as the education of stromal cells by infiltrating tumor cells, an important step in metastatic colonization, as preventing de novo niche formation represents a strategy in treating of metastatic disease [197]. Moreover, SCNs state C target scenarios where ASCs are rare and, in these cases, ASCs are tightly regulated via direct cell-cell interactions (and highly conserved cell adhesion receptors [198];) and via the molecular signals emitting from the niche [199]. Typical examples are the vertebrates’ tissue-specific SCNs, where a very tight and spatially localized control mechanisms prevent other ASC and their progenitors to participate (e.g., [200]). The colonial ascidian transient niche depicts a special case where the whole soma is regularly replaced on a weekly basis [79, 148], necessitating the continuous recurring of SCNs. State B SCNs embody statuses where ASC numbers go beyond a capacity threshold to create distinct SCNs and regulation of stemness statuses is systematically orchestrated (at the tissue level such in *Hydra* (e.g., [77, 89])) or the whole animal level, such as in flatworms (e.g., [116, 201]). Both B and A SCN states confine cases associated with high regeneration power (up to whole-body regeneration; e.g., [80, 124, 155, 202]), with the capacity of bodily fission and where the germ line is not sequestered (no need for specific somatic or germ niche sites). The state A SCNs (part of the sponge ASCs and the allogeneic tumor cells) further exemplify stemness statuses where each one of the ASCs sustains its own self-regulation network.

As specified, the three SCN stages, while distinctively presenting various architectures and properties (Table 1, Fig. 3), may reveal only a portion of the SCN repertoire existing among taxa (not yet evaluated), or even within the same animal, altogether suggesting evolutionary highly evolved SCN assemblies. Even the model organisms present SCN prototypes that differ, within the same organism, from the well-studied SCN type A, Ecdysozoan midgut ASCs as the mammalians muscle satellite cells, brain stem cells, liver stem cells, and mesenchymal stem cells do not follow the SCN type A paradigm of the bone marrow and ecydsozoan germ SCNs (e.g., [69, 203, 204]). In the same way, archaeocytes in Demospongiae [173] fall within the ASC state A domain, and choanocytes in the Hexactinellida [165] would be included within the B ASC state.

## Future perspectives

Here we review SCNs through the lens of “model vertebrates” and ecdysozoan “model invertebrates” together with non-model marine/aquatic invertebrates. From its inception, the term “SC niche” [9] has highlighted this “entity” as an extremely confined environmental (anatomical) site that supports the maintenance and eventual differentiation of SCs. This conception, which was based on hematopoietic system studies, was further supported by results obtained from SCNs in other organs and various model organisms, all of which appeared, at onset, to be surprisingly simple in structure [15]. With time, the “niche” concept has evolved to include the cellular components of the microenvironment surrounding stem cells and the molecular and hormonal signals emanating from various cells that interact with and regulate the stem cells.

To better understand the components involved in the normal physiology of niches, it becomes necessary to widen our knowledge of where and how SCs reside and interact with their microenvironment. Such an approach would imply sampling of additional, as yet unstudied taxa, from which SCNs will have to be described. The literature on SCNs in additional aquatic and marine organisms that has been accumulated over the last years help illuminate modes of SCN types, structures, and organizations that differ from those revealed from the established “model” systems. Based on the SCN descriptions provided in this manuscript, it is evident that our current understanding of the structure and function of SCNs across the animal kingdom is still very fragmentary, with open questions regarding niche architectures that may result from our limitations in experimental approaches (such as identifying the stem cells) and the limited numbers of taxa studied. Following the proposed conceptual notion that SCNs are shaped by natural selection forces that amalgamate niche architectures and constructions along a continuum of structural/functional properties, we envision three scientific approaches to study the structural/functional properties presented by various SCNs:

1. *The architecture/state of the niche.* The spatial relationships of cells, tissues, and ECM components around harboring stem cells in a specific niche need to be mapped in detail. Certainly not all components (or cells) in the neighborhood would be active components of the niche, though we should define what are the 3D disposition of those (when existing) before elucidating how they are interacting with the stem cells. A putative avenue that some of us are using is to perform 3D reconstruction using (automated) serial TEM methods in stem cell homing areas ([205]; Martinez, unpublished). The procedures allow cellular (and subcellular) resolutions and detailed mapping of cells and cell interactions within the “putative” niche.
2. *The molecular components of the niche*. The classical methodologies employed for uncovering molecular players within SCNs include mutagenesis, immunochemical, and in situ hybridization. Yet, culturing of stem cells outside the organism provides a new avenue for testing candidate genes and gene products, whether using targeted candidates, generalized molecular screens of single-cell transcriptomes. Nowadays, the major stumbling block is the lack of cell cultures and cell lines from either marine/aquatic invertebrate species [206], and in particular, the lack of any stem cell culture. While promising recent avenues, like the primary cultures of planarian neoblasts (Lei et al, 2019, Biorxiv; preprint), are encouraging, it should be noted that in vitro approaches are incapable in recapitulating the temporal and spatial niche signaling. In vertebrate models, this obstacle is being addressed by the use of new cell culture methodologies that employ microfabrication, with microfluidics and photolithography as major developments. The encapsulation of stem cells in miniaturized structures allows the configuration of high-throughput analyses of stem cell niche candidates, like regulatory factors (reviewed in [207, 208]), and the opportunity to engineer and control individual niche components, further multiplexing by hybrid devices that simultaneously provide macroscopic and microscopic control over the niche and the stem cells fate.

A related approach relies on employing 3D culture systems (collectively called spheroids), which better mirror in vivo situations. Spheroids further allow an improved characterization of factors crucially acting at microenvironment levels, the roles of culture compositions and conditions in the maintenance of stem cell populations, thus allowing the follow-up of gene expression in different areas of the spheroid (e.g., [209]).

3- *Expression profiling with spatial resolution.* The combination of single-cell transcriptomics and the fine-resolution mapping of gene profiles (or clusters) in different cell types within a tissue (termed Spatial Transcriptomics) allows an unprecedented analysis of spatial domains of expression of many genes. Of particular relevance is the use of spatial transcriptomics in fetal tissues, where stem cells and their neighbors are recognized by specific gene expression profiles [210]. An alternative to spatial transcriptomics is the use of partially dissociated cells, followed by multiplet sequencing (CIM-seq), a method that allows to identify expression profiles with single-cell resolution after computational deconvolution of profiles associated to these groups of cells. This approach has successfully identified the molecular landscape of stem neighboring cells [211].

The above technologies, mostly employed in mammalian studies, can be added to the methodological tool-box for elucidating the architectural disposition and gene regulation, in space and time, of ASCs in aquatic/marine invertebrates. Yet, any particular study should also consider the variable morphological structures, as well as the different life strategies represented by the wide range of marine invertebrates, that may further bear conceptual and methodological limitations for clarifying the full account of SCN types. The literature already attests (e.g., [5]) that SCNs are configured differently in many invertebrate taxa and are dramatically unalike to the vertebrates. In many aquatic and marine environments, animals commonly utilize ASCs as “regenerative building blocks” for coping with hostile environments or for a wide range of asexual reproduction strategies, integrating the commonly available ASCs as participants in addition to the long-established homeostasis functions. This most probably contributed to the less constrained and structurally defined SCNs in so many marine/aquatic invertebrates.

## Data Availability

Not applicable.

## References

[1] Blau HM, Brazelton TR, Weimann JM (2001). The evolving concept of a stem cell: Entity or function?. Cell.

[2] Lanza R, Gearhart J, Hogan B, Melton D, Pedersen R, Thomas ED, Thomson J, Wilmut I (2009). Essentials of Stem Cell Biology.

[3] Morrison SJ, Shah NM, Anderson DJ (1997). Regulatory mechanisms in stem cell biology. Cell.

[4] Post Y, Clevers H (2019). Defining adult stem cell function at its simplest: the ability to replace lost cells through mitosis. Cell Stem Cell.

[5] Rinkevich B, Ballarin L, Martinez P, Somorjai I, Ben-Hamo O, Borisenko I, et al. A pan-metazoan concept for adult stem cells: the wobbling Penrose landscape. Biol Rev. 2022;97(1):299–325. 10.1111/brv.12801 PMC9292022 34617397

[6] Weissman IL (2000). Stem cells: Units of development, units of regeneration, and units in evolution. Cell.

[7] Zakrzewski W, Dobrzyński M, Szymonowicz M, Rybak Z (2019). Stem cells: past, present, and future. Stem Cell Res Ther.

[8] Cheung TH, Rando TA (2013). Molecular regulation of stem cell quiescence. Nat Rev Mol Cell Biol..

[9] Schofield R (1978). The relationship between the spleen colony-forming cell and the haemopoietic stem cell. Blood Cells.

[10] Lander AD, Kimble J, Clevers H, Fuchs E, Montarras D, Buckingham M, Calof AL, Trumpp A, Oskarsson T (2012). What does the concept of the stem cell niche really mean today?. BMC Biology.

[11] Li X, Zeng X, Xu Y, Wang B, Zhao Y, Lai X, Qian P, Huang H (2020). Mechanisms and rejuvenation strategies for aged hematopoietic stem cells. J Hematol Oncol..

[12] Shim J, Gururaja-Rao S, Banerjee U. Nutritional regulation of stem and progenitor cells in Drosophila. Development (Cambridge). 2013. 10.1242/dev.079087. 10.1242/dev.079087 PMC3833425 24255094

[13] Mazo IB, Massberg S, von Andrian UH (2011). Hematopoietic stem and progenitor cell trafficking. Trends Immunol..

[14] Mikkola HKA, Orkin SH (2006). The journey of developing hematopoietic stem cells. Development..

[15] Ohlstein B, Kai T, Decotto E, Spradling A (2004). The stem cell niche: Theme and variations. Curr Opin Cell Biol..

[16] Alié A, Leclère L, Jager M, Dayraud C, Chang P, Le Guyader H, Quéinnec E, Manuel M (2011). Somatic stem cells express Piwi and Vasa genes in an adult ctenophore: ancient association of ‘germline genes’ with stemness. Dev Biol..

[17] Beltz BS, Zhang Y, Benton JL, Sandeman DC (2011). Adult neurogenesis in the decapod crustacean brain: a hematopoietic connection?. Eur J Neurosci.

[18] Giani VC, Yamaguchi E, Boyle MJ, Seaver EC (2011). Somatic and germline expression of piwi during development and regeneration in the marine polychaete annelid Capitella teleta. EvoDevo.

[19] Li L, Xie T (2005). Stem cell niche: structure and function. Annu Rev Cell Dev Biol..

[20] Bosch TCG (2009). Hydra and the evolution of stem cells. BioEssays.

[21] Funayama N (2018). The cellular and molecular bases of the sponge stem cell systems underlying reproduction, homeostasis and regeneration. Int J Dev Biol..

[22] Laird DJ, De Tomaso AW, Weissman IL (2005). Stem cells are units of natural selection in a colonial ascidian. Cell..

[23] Nakanishi N, Camara AC, Yuan DC, Gold D a, Jacobs DK (2015). Gene Expression Data from the Moon Jelly, Aurelia, Provide insights into the evolution of the combinatorial code controlling animal sense organ development. Plos One.

[24] Rinkevich Y, Voskoboynik A, Rosner A, Rabinowitz C, Paz G, Oren M, Douek J, Alfassi G, Moiseeva E, Ishizuka KJ, Palmeri KJ, Weissman IL, Rinkevich B (2013). Repeated, long-term cycling of putative stem cells between niches in a basal chordate. Dev Cell.

[25] Voskoboynik A, Soen Y, Rinkevich Y, Rosner A, Ueno H, Reshef R, Ishizuka KJ, Palmeri KJ, Moiseeva E, Rinkevich B, Weissman IL (2008). Identification of the endostyle as a stem cell niche in a colonial chordate. Cell Stem Cell.

[26] De Mulder K, Kuales G, Pfister D, Willems M, Egger B, Salvenmoser W, Thaler M, Gorny AK, Hrouda M, Borgonie G, Ladurner P (2009). Characterization of the stem cell system of the acoel Isodiametra pulchra. BMC Dev Biol..

[27] Juliano C, Wang J, Lin H (2011). Uniting germline and stem cells: the function of piwi proteins and the piRNA pathway in diverse organisms. Annu Rev Genet.

[28] Rinkevich Y, Rosner A, Rabinowitz C, Lapidot Z, Moiseeva E, Rinkevich B (2010). Piwi positive cells that line the vasculature epithelium, underlie whole body regeneration in a basal chordate. Dev Biol..

[29] Vogel G (2003). STEM CELLS: “Stemness” Genes Still Elusive. Science.

[30] Rosner A, Paz G, Rinkevich B (2006). Divergent roles of the DEAD box protein BS-PL10, the urochordate homologue of human DDX3 and DDX3Y proteins in colony astogeny and ontogeny. Dev Dyn.

[31] Pfister D, De Mulder K, Hartenstein V, Kuales G, Borgonie G, Marx F, Morris J, Ladurner P (2008). Flatworm stem cells and the germ line: developmental and evolutionary implications of macvasa expression in Macrostomum lignano. Dev Biol..

[32] Rosner A, Moiseeva E, Rinkevich Y, Lapidot Z, Rinkevich B (2009). Vasa and the germ line lineage in a colonial urochordate. Dev Biol.

[33] Wang Z, Lin H (2004). Nanos maintains germline stem cell self-renewal by preventing differentiation. Science..

[34] Rosner A, Moiseeva E, Rabinowitz C, Rinkevich B (2013). Germ lineage properties in the urochordate Botryllus schlosseri - From markers to temporal niches. Dev Biol.

[35] Rebscher N (2014). Establishing the germline in spiralian embryos. Int J Dev Biol..

[36] Bolker JA (1995). Model systems in developmental biology. BioEssays.

[37] Trumpp A, Essers M, Wilson A (2010). Awakening dormant haematopoietic stem cells. Nat Rev Immunol..

[38] Medvinsky A, Rybtsov S, Taoudi S (2011). Embryonic origin of the adult hematopoietic system: advances and questions. Development..

[39] Crane GM, Jeffery E, Morrison SJ (2017). Adult haematopoietic stem cell niches. Nat Rev Immunol.

[40] Eliazer S, Buszczak M (2011). Finding a niche: studies from the Drosophila ovary. Stem Cell Res Ther..

[41] Hanna CB, Hennebold JD (2014). Ovarian germline stem cells: an unlimited source of oocytes?. Fertil Steril..

[42] Huang D, Chen C, Hao X, Gu H, Xie L, Yu Z, Zheng J (2019). Metabolic regulations in hematopoietic stem cells. Adv Exp Med Biol..

[43] Lee HJ, Li N, Evans SM, Diaz MF, Wenzel PL (2013). Biomechanical force in blood development: extrinsic physical cues drive pro-hematopoietic signaling. Differentiation..

[44] Lucas D (2019). Leukocyte trafficking and regulation of murine hematopoietic stem cells and their niches. Front Immunol..

[45] Oatley JM, Brinster RL (2012). The germline stem cell niche unit in mammalian testes. Physiol Rev.

[46] Guo J, Sosa E, Chitiashvili T, Nie X, Rojas EJ, Oliver E, Plath K, Hotaling JM, Stukenborg JB, Clark AT, Cairns BR, DonorConnect (2021). Single-cell analysis of the developing human testis reveals somatic niche cell specification and fetal germline stem cell establishment. Cell Stem Cell.

[47] Hardy RW, Tokuyasu KT, Lindsley DL, Garavito M (1979). The germinal proliferation center in the testis of Drosophila melanogaster. J Ultrastruct Res..

[48] Xie T, Spradling AC (2000). A niche maintaining germ line stem cells in the Drosophila ovary. Science..

[49] Hayashi Y, Yoshinari Y, Kobayashi S, Niwa R (2020). The regulation of Drosophila ovarian stem cell niches by signaling crosstalk. Curr Opin Insect Sci..

[50] Chen D, McKearin D (2005). Gene circuitry controlling a stem cell niche. Curr Biol..

[51] Wang X, Page-McCaw A (2014). A matrix metalloproteinase mediates long-distance attenuation of stem cell proliferation. J Cell Biol..

[52] Jevitt A, Chatterjee D, Xie G, Wang XF, Otwell T, Huang YC, Deng WM (2020). A single-cell atlas of adult Drosophila ovary identifies transcriptional programs and somatic cell lineage regulating oogenesis. PLoS Biol..

[53] Rust K, Byrnes LE, Yu KS, Park JS, Sneddon JB, Tward AD, Nystul TG (2020). A single-cell atlas and lineage analysis of the adult Drosophila ovary. Nat Commun.

[54] Slaidina M, Banisch TU, Gupta S, Lehmann RA (2020). A single-cell atlas of the developing Drosophila ovary identifies follicle stem cell progenitors. Genes Dev.

[55] Shi Z, Lim C, Tran V, Cui K, Zhao K, Chen X (2020). Single-cyst transcriptome analysis of Drosophila male germline stem cell lineage. Development.

[56] Hubbard EJA, Schedl T (2019). Biology of the Caenorhabditis elegans Germline Stem Cell System. Genetics..

[57] Joshi PM, Riddle MR, Djabrayan NJV, Rothman JH (2010). Caenorhabditis elegans as a model for stem cell biology. Dev Dyn..

[58] Kimble J (1981). Alterations in cell lineage following laser ablation of cells in the somatic gonad of Caenorhabditis elegans. Dev Biol..

[59] Cinquin O, Crittenden SL, Morgan DE, Kimble J (2010). Progression from a stem cell-like state to early differentiation in the C. elegans germ line. Proc Natl Acad Sci U S A..

[60] Austin J, Kimble J (1987). glp-1 Is required in the germ line for regulation of the decision between mitosis and meiosis in C. elegans. Cell..

[61] Gazave E, Lapébie P, Richards GS, Brunet F, Ereskovsky AV, Degnan BM, Borchiellini C, Vervoort M, Renard E (2009). Origin and evolution of the Notch signalling pathway: an overview from eukaryotic genomes. BMC Evol Biol.

[62] Koch U, Lehal R, Radtke F (2013). Stem cells living with a Notch. Development (Cambridge)..

[63] Van Hoffelen S, Herman MA (2006). Stem cells: specifying stem-cell niches in the worm. Curr Biol..

[64] Gordon KL, Zussman JW, Li X, Miller C, Sherwood DR (2020). Stem cell niche exit in C. elegans via orientation and segregation of daughter cells by a cryptic cell outside the niche. Elife.

[65] Persico V, Callaini G, Riparbelli MG (2019). The male stem cell niche of Drosophila melanogaster: interactions between the germline stem cells and the hub. Exp Cell Res.

[66] Schmidt ED, Dorn A (2004). Structural polarity and dynamics of male germline stem cells in the milkweed bug (Oncopeltus fasciatus). Cell Tissue Res..

[67] Packer JS, Zhu Q, Huynh C, Sivaramakrishnan P, Preston E, Dueck H, Stefanik D, Tan K, Trapnell C, Kim J, Waterston RH, Murray JI (2019). A lineage-resolved molecular atlas of C. elegans embryogenesis at single-cell resolution. Science.

[68] Schild ES, Mars J, Ebbing A, Vivié J, Betist M, Korswagen HC (2021). Spatial transcriptomics of the nematode Caenorhabditis elegans using RNA tomography. STAR Protoc.

[69] Jones DL, Wagers AJ (2008). No place like home: anatomy and function of the stem cell niche. Nat Rev Mol Cell Biol.

[70] Pagella P, Neto E, Lamghari M, Mitsiadis TA (2015). Investigation of orofacial stem cell niches and their innervation through microfluidic devices. Eur Cell Mater.

[71] Kilian KA, Bugarija B, Lahn BT, Mrksich M (2010). Geometric cues for directing the differentiation of mesenchymal stem cells. Proc Natl Acad Sci U S A..

[72] Appeltans W, Ahyong ST, Anderson G, Angel MV, Artois T, Bailly N (2012). The magnitude of global marine species diversity. Curr Biol..

[73] Knope ML, Bush AM, Frishkoff LO, Heim NA, Payne JL (2020). Ecologically diverse clades dominate the oceans via extinction resistance. Science.

[74] Williamson M, Ormond M, Gage RFG, Angel JD (1997). Marine biodiversity in its global context. Marine Biodiversity: Patterns and Processes.

[75] Rink JC (2013). Stem cell systems and regeneration in planaria. Dev Genes Evol.

[76] Benton JL, Kery R, Li J, Noonin C, Söderhäll I, Beltz BS (2014). Cells from the immune system generate adult-born neurons in crayfish. Dev Cell.

[77] Bosch TCG, Anton-Erxleben F, Hemmrich G, Khalturin K (2010). The hydra polyp: nothing but an active stem cell community. Dev Growth Differ.

[78] Lindsay-Mosher N, Chan A, Pearson BJ (2020). Planarian EGF repeat-containing genes megf6 and hemicentin are required to restrict the stem cell compartment. PLoS Genetics.

[79] Voskoboynik A, Simon-Blecher N, Soen Y, Rinkevich B, De Tomaso AW, Ishizuka KJ, Weissman IL (2007). Striving for normality: whole body regeneration through a series of abnormal zooidal generations. FASEB J.

[80] Vogg MC, Galliot B, Tsiairis CD (2019). Model systems for regeneration: Hydra. Development.

[81] Frank U, Nicotra ML, Schnitzler CE (2020). The colonial cnidarian Hydractinia. Evodevo.

[82] Amiel AR, Johnston HT, Nedoncelle K, Warner JF, Ferreira S, Röttinger E (2015). Characterization of morphological and cellular events underlying oral regeneration in the sea anemone, Nematostella vectensis. Int J Mol Sci.

[83] Gold DA, Jacobs DK (2013). Stem cell dynamics in Cnidaria: are there unifying principles?. Dev Genes Evol.

[84] Bosch TCG, David CN (1987). Stem cells of Hydra magnipapillata can differentiate into somatic cells and germ line cells. Dev Biol.

[85] Gahan JM, Bradshaw B, Flici H, Frank U (2016). The interstitial stem cells in Hydractinia and their role in regeneration. Curr Opin Genet Dev..

[86] Schmid V (1992). Transdifferentiation in Medusae. Int Rev Cytol..

[87] Künzel T, Heiermann R, Frank U, Müller W, Tilmann W, Bause M, Nonn A, Helling M, Schwarz RS, Plickert G (2010). Migration and differentiation potential of stem cells in the cnidarian Hydractinia analysed in eGFP-transgenic animals and chimeras. Dev Biol..

[88] Müller WA, Teo R, Frank U (2004). Totipotent migratory stem cells in a hydroid. Dev Biol..

[89] Bode HR (1996). The interstitial cell lineage of hydra: a stem cell system that arose early in evolution. J Cell Sci.

[90] David CN, Plotnick I (1980). Distribution of interstitial stem cells in Hydra. Dev Biol..

[91] Hobmayer B, Jenewein M, Eder D, Eder MK, Glasauer S, Gufler S, Hartl M, Salvenmoser W (2012). Stemness in Hydra - a current perspective. Int J Dev Biol..

[92] Boehm AM, Bosch TCG (2012). Migration of multipotent interstitial stem cells in Hydra. Zoology..

[93] Hartl M, Mitterstiller AM, Valovka T, Breuker K, Hobmayer B, Bister K (2010). Stem cell-specific activation of an ancestral myc protooncogene with conserved basic functions in the early metazoan Hydra. Proc Natl Acad Sci U S A..

[94] Käsbauer T, Towb P, Alexandrova O, David CN, Dall'armi E, Staudigl A, Stiening B, Böttger A (2007). The Notch signaling pathway in the cnidarian Hydra. Dev Biol..

[95] Siebert S, Farrell JA, Cazet JF, Abeykoon Y, Primack AS, Schnitzler CE, Juliano CE (2019). Stem cell differentiation trajectories in Hydra resolved at single-cell resolution. Science.

[96] David CN (2012). Interstitial stem cells in Hydra: multipotency and decision-making. Int J Dev Biol..

[97] Hobmayer B, Rentzsch F, Kuhn K, Happel CM, von Laue CC, Snyder P, Rothbächer U, Holstein TW (2000). WNT signalling molecules act in axis formation in the diploblastic metazoan Hydra. Nature..

[98] Duffy DJ, Plickert G, Kuenzel T, Tilmann W, Frank U (2010). Wnt signaling promotes oral but suppresses aboral structures in Hydractinia metamorphosis and regeneration. Development..

[99] Hartl M, Glasauer S, Gufler S, Raffeiner A, Puglisi K, Breuker K, Bister K, Hobmayer B (2019). Differential regulation of myc homologs by Wnt/β-Catenin signaling in the early metazoan Hydra. FEBS J..

[100] Khalturin K, Anton-Erxleben F, Milde S, Plötz C, Wittlieb J, Hemmrich G, Bosch TCG (2007). Transgenic stem cells in Hydra reveal an early evolutionary origin for key elements controlling self-renewal and differentiation. Dev Biol..

[101] Teo R, Möhrlen F, Plickert G, Müller WA, Frank U (2006). An evolutionary conserved role of Wnt signaling in stem cell fate decisions. Dev Biol..

[102] Holstein TW, David CN (1990). Putative intermediates in the nerve cell differentiation pathway in Hydra have properties of multipotent stem cells. Dev Biol.

[103] Buzgariu W, Al Haddad S, Tomczyk S, Wenger Y, Galliot B (2015). Multi-functionality and plasticity characterize epithelial cells in Hydra. Tissue Barriers.

[104] Fujita S, Kuranaga E, Nakajima YI (2021). Regeneration potential of jellyfish: cellular mechanisms and molecular insights. Genes (Basel).

[105] Kirillova A, Genikhovich G, Pukhlyakova E, Demilly A, Kraus Y, Technau U (2018). Germ-layer commitment and axis formation in sea anemone embryonic cell aggregates. Proc Natl Acad Sci U S A..

[106] Raz-Bahat M, Erez J, Rinkevich B (2006). In vivo light-microscopic documentation for primary calcification processes in the hermatypic coral Stylophora pistillata. Cell Tissue Res..

[107] Sanders SM, Ma Z, Hughes JM, Riscoe BM, Gibson GA, Watson AM, Flici H, Frank U, Schnitzler CE, Baxevanis AD, Nicotra ML (2018). CRISPR/Cas9-mediated gene knockin in the hydroid Hydractinia symbiolongicarpus. BMC Genomics..

[108] Klimovich A, Wittlieb J, Bosch TCG (2019). Transgenesis in Hydra to characterize gene function and visualize cell behavior. Nat Protoc..

[109] Quiroga-Artigas G, Duscher A, Lundquist K, Waletich J, Schnitzler CE (2020). Gene knockdown via electroporation of short hairpin RNAs in embryos of the marine hydroid Hydractinia symbiolongicarpus. Sci Rep.

[110] Achatz JG, Chiodin M, Salvenmoser W, Tyler S, Martinez P (2013). The Acoela: on their kind and kinships, especially with nemertodermatids and xenoturbellids (Bilateria incertae sedis). Org Divers Evol.

[111] Baguñà J, Salo E, Auladell C (1989). Regeneration and pattern formation in planarians. III. Evidence that neoblasts are totipotent stem cells and the source of blastema cells. Development.

[112] Bely AE, Sikes JM (2010). Acoel and platyhelminth models for stem-cell research. J Biol.

[113] Mouton S, Wudarski J, Grudniewska M, Berezikov E (2018). The regenerative flatworm macrostomum lignano, a model organism with high experimental potential. Int J Dev Biol.

[114] Rozario T, Quinn EB, Wang J, Davis RE, Newmark PA (2018). Region-specific regulation of stem cell-driven regeneration in tapeworms. Elife.

[115] Rink JC (2018). Stem Cells, Patterning and regeneration in planarians: Self-Organization at the Organismal Scale. Methods Mol Biol..

[116] Baguñà J, Bishop B, Hall CD (2020). Planarian neoblasts. Nondeferred, Multipurpose stem cells for body homeostasis, growth, degrowth, and regeneration. Deferring Development. Setting Aside Cells for Future Use in Development and Evolution.

[117] Molina MD, Cebrià F (2021). Decoding stem cells: an overview on planarian stem cell heterogeneity and lineage progression. Biomolecules.

[118] Lange CS (1967). A quantitative study of the number and distribution of neoblasts in Dugesia lugubris (Planaria) with reference to size and ploidy. J Embryol Exp Morphol..

[119] Collins JJ, Wang B, Lambrus BG, Tharp ME, Iyer H, Newmark PA (2013). Adult somatic stem cells in the human parasite Schistosoma mansoni. Nature..

[120] Baguñá J, Romero R (1981). Quantitative analysis of cell types during growth, degrowth and regeneration in the planarians Dugesia mediterranea and Dugesia tigrina. Hydrobiologia..

[121] Sánchez Alvarado A (2007). Stem cells and the Planarian Schmidtea mediterranea. C R Biologies..

[122] Orii H, Sakurai T, Watanabe K (2005). Distribution of the stem cells (neoblasts) in the planarian Dugesia japonica. Dev Genes Evol..

[123] Srivastava M, Mazza-Curll KL, van Wolfswinkel JC, Reddien PW (2014). Whole-body acoel regeneration is controlled by Wnt and Bmp-Admp signaling. Curr Biol..

[124] Gehrke AR, Srivastava M (2016). Neoblasts and the evolution of whole-body regeneration. Curr Opin Genet Dev.

[125] Baguñà J (2012). The planarian neoblast: the rambling history of its origin and some current black boxes. Int J Dev Biol..

[126] Wagner DE, Wang IE, Reddien PW (2011). Clonogenic neoblasts are pluripotent adult stem cells that underlie planarian regeneration. Science..

[127] Eisenhoffer GT, Kang H, Sánchez AA (2008). Molecular analysis of stem cells and their descendants during cell turnover and regeneration in the planarian Schmidtea mediterranea. Cell Stem Cell..

[128] Labbé RM, Irimia M, Currie KW, Lin A, Zhu SJ, Brown DD, Ross EJ, Voisin V, Bader GD, Blencowe BJ, Pearson BJ (2012). A comparative transcriptomic analysis reveals conserved features of stem cell pluripotency in planarians and mammals. Stem Cells..

[129] Wagner DE, Ho JJ, Reddien PW (2012). Genetic regulators of a pluripotent adult stem cell system in planarians identified by RNAi and clonal analysis. Cell Stem Cell..

[130] Zeng A, Li H, Guo L, Gao X, McKinney S, Wang Y, Yu Z, Park J, Semerad C, Ross E, Cheng LC, Davies E, Lei K, Wang W, Perera A, Hall K, Peak A, Box A, Sánchez AA (2018). Prospectively isolated tetraspanin + neoblasts are adult pluripotent stem cells underlying planarian regeneration. Cell.

[131] Raz AA, Wurtzel O, Reddien PW (2021). Planarian stem cells specify fate yet retain potency during the cell cycle. Cell Stem Cell..

[132] Forsthoefel DJ, James NP, Escobar DJ, Stary JM, Vieira AP, Waters FA, Newmark PA (2012). An RNAi screen reveals intestinal regulators of branching morphogenesis, differentiation, and stem cell proliferation in planarians. Dev Cell..

[133] Chan A, Ma S, Pearson BJ, Chan D (2021). Collagen IV differentially regulates planarian stem cell potency and lineage progression. Proc Natl Acad Sci U S A.

[134] Oviedo NJ, Levin M (2007). smedinx-11 is a planarian stem cell gap junction gene required for regeneration and homeostasis. Development.

[135] Baguñà J, Saló E, Romero R (1989). Effects of activators and antagonists of the neuropeptides substance P and substance K on cell proliferation in planarians. Int J Dev Biol..

[136] Barberán S, Fraguas S, Cebrià F (2016). The EGFR signaling pathway controls gut progenitor differentiation during planarian regeneration and homeostasis. Development.

[137] Lapan SW, Reddien PW (2012). Transcriptome analysis of the planarian eye identifies ovo as a specific regulator of eye regeneration. Cell Rep..

[138] Gschwentner R, Ladurner P, Nimeth K, Rieger R (2001). Stem cells in a basal bilaterian. Cell Tissue Res.

[139] Duruz J, Kaltenrieder C, Ladurner P, Bruggmann R, Martìnez P, Sprecher SG (2021). Acoel Single-Cell Transcriptomics: Cell type analysis of a deep branching bilaterian. Mol Biol Evol..

[140] Rossi L, Salvetti A (2019). Planarian stem cell niche, the challenge for understanding tissue regeneration. Semin Cell Dev Biol.

[141] Newmark PA, Sánchez Alvarado A (2000). Bromodeoxyuridine specifically labels the regenerative stem cells of planarians. Dev Biol..

[142] Durant F, Lobo D, Hammelman J, Levin M (2016). Physiological controls of large-scale patterning in planarian regeneration: a molecular and computational perspective on growth and form. Regeneration (Oxf)..

[143] Levin M, Pietak AM, Bischof J (2019). Planarian regeneration as a model of anatomical homeostasis: recent progress in biophysical and computational approaches. Semin Cell Dev Biol..

[144] González-Estévez C, Felix DA, Smith MD, Paps J, Morley SJ, James V, Sharp TV, Aboobaker AA (2012). SMG-1 and mTORC1 act antagonistically to regulate response to injury and growth in planarians. PLoS Genet.

[145] Issigonis M, Newmark PA (2019). From worm to germ: germ cell development and regeneration in planarians. Curr Top Dev Biol..

[146] Issigonis M, Redkar A, Rozario T, Khan U, Mejia-Sanchez R, Lapan S, et al. Krüppel-like factor 4 is required for development and regeneration of germline and yolk cells from somatic stem cells in planarians. bioRxiv. 2021. 10.1101/2021.11.08.467675. 10.1371/journal.pbio.3001472 PMC9286257 35839223

[147] Manni L, Anselmi C, Cima F, Gasparini F, Voskoboynik A, Martini M, Peronato A, Burighel P, Zaniolo G, Ballarin L (2019). Sixty years of experimental studies on the blastogenesis of the colonial tunicate Botryllus schlosseri. Dev Biol.

[148] Rinkevich B (2002). The colonial urochordate Botryllus schlosseri: from stem cells and natural tissue transplantation to issues in evolutionary ecology. BioEssays.

[149] Rosental B, Kowarsky M, Seita J, Corey DM, Ishizuka KJ, Palmeri KJ, et al. Complex mammalian-like haematopoietic system found in a colonial chordate. Nature. 2018;564(7736):425–9. 10.1038/s41586-018-0783-x. London: Springer Nature. 10.1038/s41586-018-0783-x PMC6347970 30518860

[150] Ogasawara M, Di Lauro R, Satoh N (1999). Ascidian homologs of mammalian thyroid transcription factor-1 gene are expressed in the endostyle. Zool Sci..

[151] Ben-Hamo O, Rosner A, Rabinowitz C, Oren M, Rinkevich B (2018). Coupling astogenic aging in the colonial tunicate Botryllus schlosseri with the stress protein mortalin. Dev Biol..

[152] Magor BG, De Tomaso AW, Rinkevich B, Weissman IL (1999). Allorecognition in colonial tunicates: protection against predatory cell lineages?. Immunol Rev.

[153] Blanchoud S, Rinkevich B, Wilson MJ. Whole-body regeneration in the colonial tunicate Botrylloides leachii. In: Kloc J, Kubiac M, editors. Marine Organisms as Model Systems in Biology and Medicine: Springer; 2018. p. 337–55. 10.1007/978-3-319-92486-1_16 30083927

[154] Kassmer SH, Rodriguez D, De Tomaso AW (2020). Evidence that ABC transporter-mediated autocrine export of an eicosanoid signaling molecule enhances germ cell chemotaxis in the colonial tunicate Botryllus schlosseri. Development (Cambridge, England).

[155] Rinkevich Y, Paz G, Rinkevich B, Reshef R (2007). Systemic bud induction and retinoic acid signaling underlie whole body regeneration in the urochordate Botrylloides leachi. PLoS Biology..

[156] Auger H, Sasakura Y, Joly JS, Jeffery WR (2010). Regeneration of oral siphon pigment organs in the ascidian Ciona intestinalis. Dev Biol..

[157] Jeffery WR (2015). Regeneration, stem cells, and aging in the tunicate Ciona: insights from the oral siphon. Int Rev Cell Mol Biol..

[158] Jeffery WR (2019). Progenitor targeting by adult stem cells in Ciona homeostasis, injury, and regeneration. Dev Biol.

[159] Jiménez-Merino J, Santos De Abreu I, Hiebert LS, Allodi S, Tiozzo S, De Barros CM, Brown FD (2019). Putative stem cells in the hemolymph and in the intestinal submucosa of the solitary ascidian Styela plicata. EvoDevo..

[160] Funayama N (2013). The stem cell system in demosponges: suggested involvement of two types of cells: archeocytes (active stem cells) and choanocytes (food-entrapping flagellated cells). Dev Genes Evol..

[161] Leys SP, Mackie GO, Reiswig HM. The Biology of Glass Sponges. Adv Marine Biol. 2007;52:1–145. 10.1016/S0065-2881(06)52001-2. Amsterdam: Elsevier B.V. 10.1016/S0065-2881(06)52001-2 17298890

[162] Ijima I (1901). Studies on the Hexactinelida, contribution I. (Euplectellidae). J Coll Sci Imper Univ Tokyo.

[163] Singla C, Mackie MG. Studies on hexactinellid sponges. I. Histology of Rhabdocalyptus dawsoni (Lambe, 1873). Phil Trans Roy Soc Lond. 1983;301:365–400. 10.1098/rstb.1983.0028.

[164] Alexander BE, Liebrand K, Osinga R, van der Geest HG, Admiraal W, Cleutjens JP, Schutte B, Verheyen F, Ribes M, van Loon E, de Goeij JM (2014). Cell turnover and detritus production in marine sponges from tropical and temperate benthic ecosystems. PLoS One.

[165] Ereskovsky A, Lavrov A. Porifera. In: LaDouceur EEB, editor. Invertebrate Histology: John Wiley & Sons, Inc; 2021.

[166] Ereskovsky AV (2010). The Comparative Embryology of Sponges.

[167] Amiel AR, Foucher K, Ferreira S, Röttinger E. Synergic coordination of stem cells is required to induce a regenerative response in anthozoan cnidarians. BioRxiv. 2019. 10.1101/2019.12.31.891804.

[168] Ramon-Mateu J, Ellison ST, Angelini TE, Martindale MQ (2019). Regeneration in the ctenophore Mnemiopsis leidyi occurs in the absence of a blastema, requires cell division, and is temporally separable from wound healing. BMC Biology.

[169] Rinkevich B (2011). Quo vadis chimerism?. Chimerism.

[170] Ujvari B, Papenfuss AT, Belov K (2016). Transmissible cancers in an evolutionary context. BioEssays..

[171] Cabarcas SM, Mathews LA, Farrar WL (2011). The cancer stem cell niche-there goes the neighborhood?. Int J Cancer.

[172] Lean C, Plutynski A (2016). The evolution of failure: explaining cancer as an evolutionary process. Biol Philos..

[173] Okamoto K, Nakatsukasa M, Alié A, Masuda Y, Agata K, Funayama N (2012). The active stem cell specific expression of sponge Musashi homolog EflMsiA suggests its involvement in maintaining the stem cell state. Mech Dev.

[174] Plaks V, Kong N, Werb Z (2015). The cancer stem cell niche: how essential is the niche in regulating stemness of tumor cells?. Cell Stem Cell..

[175] Quail DF, Joyce JA. Microenvironmental regulation of tumor progression and metastasis. Nat Med. 2013;19(11):1423–37. 10.1038/nm.3394. London: Springer Nature. 10.1038/nm.3394 PMC3954707 24202395

[176] Sneddon JB, Werb Z (2007). Location, location, location: the cancer stem cell niche. Cell Stem Cell..

[177] Di Santo JP (2008). Natural killer cells: diversity in search of a niche. Nat Immunol.

[178] Ayala-Díaz S, Medina DA, Lizano M, Manzo-Merino J (2017). Transmissible cancer: a canine transmissible venereal tumor during pregnancy, Case Report. J Cancer Res.

[179] Adamska M. Differentiation and transdifferentiation of sponge cells. In: Kloc J, Kubiak M, editors. Marine Organisms as Model Systems in Biology and Medicine: Springer International Publishing; 2018. p. 229–53. 10.1007/978-3-319-92486-1_12 30083923

[180] Lavrov AI, Kosevich IA (2016). Sponge cell reaggregation: Cellularstructure and morphogenetic potencies of multicellular aggregates. J Exp Zool A Ecol Genet Physiol.

[181] Soubigou A, Ross EG, Touhami Y, Chrismas N, Modepalli V (2020). Regeneration in the sponge Sycon ciliatum partly mimics postlarval development. Development (Cambridge, England).

[182] Shortt AJ, Secker GA, Munro PM, Khaw PT, Tuft SJ, Daniels JT (2007). Characterization of the limbal epithelial stem cell niche: novel imaging techniques permit in vivo observation and targeted biopsy of limbal epithelial stem cells. Stem Cells.

[183] Boulais PE, Frenette PS (2015). Making sense of hematopoietic stem cell niches. Blood.

[184] Alexander BE, Achlatis M, Osinga R, van der Geest HG, Cleutjens JPM, Schutte B, de Goeij JM (2015). Cell kinetics during regeneration in the sponge Halisarca caerulea: how local is the response to tissue damage?. Peer J.

[185] Lavrov AI, Bolshakov FV, Tokina DB, Ereskovsky AV (2018). Sewing up the wounds: the epithelial morphogenesis as a central mechanism of calcaronean sponge regeneration. J Exp Zool B Mol Dev Evol..

[186] Musser, J.M., Schippers, K.J., Nickel,M., Mizzon, G., Kohn, A.B., Pape, C., Hammel, J.U., Wolf, F., Liang, C., Hernandez-Plaza, A., Achim, K., Schieber, N.L., Francis, W.R., Vargas, R.S., Kling, S., Renkert, M., Feuda, R., Gaspar, I., Burkhardt, P., Bork, P. et al. (2019). Profiling cellular diversity in sponges informs animal cell type and nervous system evolution. BioRxiv. 10.1101/758276

[187] Levy S, Elek A, Grau-Bové X, Menéndez-Bravo S, Iglesias M, Tanay A, Mass T, Sebé-Pedrós A (2021). A stony coral cell atlas illuminates the molecular and cellular basis of coral symbiosis, calcification, and immunity. Cell.

[188] Fincher CT, Wurtzel O, de Hoog T, Kravarik KM, Reddien PW (2018). Cell type transcriptome atlas for the planarian Schmidtea mediterranea. Science.

[189] Li P, Nanes Sarfati D, Xue Y, Yu X, Tarashansky AJ, Quake SR, Wang B (2021). Single-cell analysis of Schistosoma mansoni identifies a conserved genetic program controlling germline stem cell fate. Nat Commun.

[190] Ferraro F, Lo Celso C, Scadden D (2010). Adult stem cells and their niches. Adv Exp Med Biol.

[191] Morrison SJ, Spradling AC (2008). Stem cells and niches: mechanisms that promote stem cell maintenance throughout life. Cell.

[192] Szade K, Gulati GS, Chan CKF, Kao KS, Miyanishi M, Marjon KD, Sinha R, George BM, Chen JY, Weissman IL (2018). Where hematopoietic stem cells live: the bone marrow niche. Antioxid Redox Signal.

[193] Lidke AK, Bannister S, Löwer AM, Apel DM, Podleschny M, Kollmann M, Ackermann CF, García-Alonso J, Raible F, Rebscher N (2014). 17β-Estradiol induces supernumerary primordial germ cells in embryos of the polychaete Platynereis dumerilii. Gen Comp Endocrinol..

[194] Schenk S, Krauditsch C, Frühauf P, Gerner C, Raible F (2016). Discovery of methylfarnesoate as the annelid brain hormone reveals an ancient role of sesquiterpenoids in reproduction. Elife..

[195] Gonzales KAU, Fuchs E (2017). Skin and its regenerative powers: an alliance between stem cells and their niche. Dev Cell..

[196] Beumer J, Clevers H (2021). Cell fate specification and differentiation in the adult mammalian intestine. Nat Rev Mol Cell Biol..

[197] Malanchi I, Santamaria-Martínez A, Susanto E, Peng H, Lehr HA, Delaloye JF, Huelsken J (2012). Interactions between cancer stem cells and their niche govern metastatic colonization. Nature.

[198] Gamulin V, Rinkevich B, Schaecke H, Kruse M, Mueller IM, Mueller WEG (1994). Cell adhesion receptors and nuclear receptors are highly conserved from the lowest metazoa (marine sponges) to vertebrates. Biol Chem Hoppe Seyler.

[199] Pennings S, Liu KJ, Qian H (2018). The stem cell niche: interactions between stem cells and their environment. Stem Cells Int.

[200] Ghosh M, Helm KM, Smith RW, Giordanengo MS, Li B, Shen H, Reynolds SD (2011). A single cell functions as a tissue-specific stem cell and the in vitro niche-forming cell. Am J Respir Cell Mol Biol.

[201] Scimone ML, Kravarik KM, Lapan SW, Reddien PW (2014). Neoblast specialization in regeneration of the planarian Schmidtea mediterranea. Stem Cell Rep..

[202] Sánchez AA (2006). Planarian regeneration: its end is its beginning. Cell..

[203] Zhang L, Theise N, Chua M, Reid LM (2008). The stem cell niche of human livers: symmetry between development and regeneration. Hepatology.

[204] Aziz A, Sebastian S, Dilworth FJ (2012). The origin and fate of muscle satellite cells. Stem Cell Rev Rep.

[205] Bery A, Cardona A, Martinez P, Hartenstein V (2010). Structure of the central nervous system of a juvenile acoel, Symsagittifera roscoffensis. Dev Genes Evol..

[206] Rinkevich B (2011). Cell cultures from marine invertebrates: new insights for capturing endless stemness. Mar Biotechnol.

[207] Kobel S, Lutolf M (2010). High-throughput methods to define complex stem cell niches. Biotechniques.

[208] Peng XY, Guo Y, Peng L, Liu J (2021). Design artificial stem cell nests for stem cell niche in a microfluidic petri dish programmed by a cell phone. Adv Mater Technol..

[209] Kim J, Adachi T (2021). Cell-fate decision of mesenchymal stem cells toward osteocyte differentiation is committed by spheroid culture. Sci Rep.

[210] Lu Y, Liu M, Yang J, Weissman SM, Pan X, Katz SG, Wang S (2021). Spatial transcriptome profiling by MERFISH reveals fetal liver hematopoietic stem cell niche architecture. Cell Discov..

[211] Andrews N, Serviss JT, Geyer N, Andersson AB, Dzwonkowska E, Šutevski I, Heijboer R, Baryawno N, Gerling M, Enge M (2021). An unsupervised method for physical cell interaction profiling of complex tissues. Nat Methods..

